# Holden on the Sphygmograph

**Published:** 1874-04

**Authors:** 


THE SPHYGMOGRAPH: *Its Physiological and Pathological indications. The Essay to which was awarded the Stevens Triennial Prize by the College of Physicians and Surgeons, New York, April,* 1873; *with* 290 *Illustrations.* By Edgar Holden, A.M., M.D., Philadelphia: Lindsay & Blakiston, 1874. Octavo; Cloth, pp. 169. Price, $3.00.

Those of our readers who are interesting themselves in the new art of medical diagnosis by Sphygmography, will find a welcome and valuable aid to their researches in the monograph before us. A cheap and very practical Sphygmographical instrument has been devised by Dr. Holden and, we believe, not patented; it is to be had in New York at a moderate price. To those who have leisure and taste for the study, it would afford delightful scientific research, as well as add to their fund of useful knowledge.

